# Genetic mapping of the ScHd1 gene in rye and an assessment of its relationship with earliness per se and plant morphology

**DOI:** 10.1007/s13353-014-0223-z

**Published:** 2014-05-20

**Authors:** Sandra Święcka, Marcin Berdzik, Beata Myśków

**Affiliations:** Department of Plant Genetics, Breeding and Biotechnology, West-Pomeranian University of Technology in Szczecin, Szczecin, Poland

**Keywords:** Flowering, Heading date, Intrinsic earliness, *Secale cereale* L, Winter rye

## Abstract

A fragment of the *ScHd1* gene derived from eight inbred lines was sequenced and showed homology to other Hd1 genes from different cereals. Sequences were analysed with respect to the presence of a single-nucleotide polymorphism (SNP) difference. A C-T transition at position 312 of the consensus sequence was found, which distinguished two lines from the remaining six. The deduced amino acid sequence revealed a high identity (93%) to a Hd1-like protein from wheat. The identified mutation allowed the localisation of *ScHd1* on a genetic map of rye (6RS). A small, statistically significant linkage between *ScHd1* and earliness per se (eps) and some morphological traits was also established. The chromosomal region, including the S76 allele for the *ScHd1* gene was linked to earlier heading, elongated spikes, a greater number of spikelets per spike and an increased weight of 1000 kernels.

Variability in flowering time in cereals depends on genes controlling photoperiod (Ppd) and vernalisation (Vrn), as well as on other genes that regulate earliness per se (eps). There are many articles concerning cereal eps loci that have been analysed and described as QTLs, but there is little published information relating to the analysis of these genes. One of the best-known eps loci is *Eps-A* ^*m*^ *1* from *Triticum monococcum* on chromosome 1A^m^ (Bullrich et al. 2002; Válarik et al. 2006; Lewis et al. 2008; Faricelli et al. 2010). The *Eps-3A* ^*m*^ gene in Einkorn wheat is also characterised quite well (Hori et al. 2007; Gawroński and Schnurbusch 2012). In rye, many QTLs determining flowering time were detected on all seven chromosomes (Myśków 2012; Myśków et al. 2012).

The object of this study was the *Hd1* gene, an orthologue of the *Arabidopsis thaliana CONSTANS* (*CO*) gene, involved in photoperiodic flowering. Hd1 gene homologues have been identified in rice, barley and wheat (Yamamoto et al. 1998; Yano et al. 2000; Kojima et al. 2002; Griffiths et al. 2003; Nemoto et al. 2003). Although homologues of *Hd1* are key regulators of photoperiodic flowering in plants of short-day (SD) zones (Cockram et al. 2007), they do not cause strong phenotypic effects in temperate cereals. However, transgenic experiments expressing the wheat gene in rice showed that it maintains its strong effect in the genetic background of SD plants (Nemoto et al. 2003).

Although the nucleotide sequence of the *Hd1* gene fragment (GU324592.1) is already known, it has not yet been mapped to the rye genome. Additionally, its role in flowering time regulation is unknown. The main purpose of this study was 1) to map *Hd1* to the rye genome; 2) to examine its role in the regulation of earliness per se; 3) to examine linkage between *ScHd1* and the distribution of morphological traits such as plant height (Ph), spike length (Sl), number of spikelets per spike (Sps), number of kernels per spike (Kps), weight of kernels per spike (Kw), thousand kernel weight (Tkw), pre-harvest sprouting (PHS) and alpha-amylase activity (AA).

Plant material included inbred lines of rye: 541, Ot1-3, Ds2, RXL10, S120, S76, C599 and 620–5, with at least 20 generations of inbreeding. These were the parental breeds used for crossbreeding to obtain high-density genetic maps (Milczarski et al. 2011, Myśków, unpublished data). In addition, the mapping populations were at our disposal (population of recombinant inbred lines: 541×Ot1-3, DS2×RXL10, S120×S76 and C599×620-2).

DNA was isolated from lyophilised leaves using the DNeasy Plant Mini Kit (Qiagen). The DNA was amplified by PCR via standard conditions (detailed methodology upon request) in a DNA Engine Dyad® Thermal Cycler (Bio-Rad Laboratories), using Thermo Scientific (Fermentas) reagents and the primer pair: Hd-F355 (5´-AGCGTGTGCGTGTCTGCGAA-3’) and Hd-R862 (5’-GCAGCCTGCCCTGCTCCTAT-3’) purchased from Genomed. Primers were designed to the conservative region containing the BBOX (B-box-type zinc finger) domain of the wheat *TaHd1* gene (AB094491.1, *TaHd1-3* mRNA for the Hd1-like protein complete coding sequence). The numbers in the primer names describe the binding positions of the primers.

The PCR amplicons were separated by electrophoresis in a 1.5% agarose gel with 1×TBE buffer, and were visualised using EtBr. Monomorphic products of amplification of ∼500 bp in length were obtained for eight analysed lines. These fragments were isolated from the gel and cloned into the pCR®II-TOPO® vector (Life Technologies, Invitrogen). A GenomeLab DTCS – Quick Start Kit (Beckman Coulter) and M13 primer were used for PCR sequencing. Sequencing was performed in a Beckman Coulter CEQ 8000 Genetic Analysis System. Each DNA strain was sequenced on both strands and a consensus sequence was obtained using BioEdit (Hall 1999) and Geneious R6 (Biomatters, http://www.geneious.com/).

The obtained sequences, ranging from 370 bp to 520 bp in length, were registered with NCBI and GenBank with the accession numbers from KJ371037 to KJ371044, consecutively. The sequences of the analysed lines and one common consensus sequence were compared with sequences deposited at the NCBI database and homology to GU324592.1 (*Secale cereale* cultivar Puma Hd1-like protein mRNA, partial cds), AB094488.1 (*Triticum aestivum* TaHd1-2 gene for Hd1-like protein, complete coding sequence), four homologous wheat sequences and to barley sequences was revealed. The percentage nucleotide identity for rye, wheat and barley was 98%, 95% and 94% (E = 0), respectively.

Sequences were analysed with respect to the presence of SNPs. Single nucleotide differences were detected at 14 sites, but they concerned a single strand, and probably resulted from amplification errors during PCR. Moreover, a single SNP (a C-T transition at position 312 of the consensus sequence) that distinguished lines S76 and C599-5 from the other six lines was detected. Using the predicted amino acid sequence to perform a similarity search using the NCBI database showed a high identity of 99% (one variant amino acid) and 93% (12 different amino acids) to rye (ADR51712.1) and wheat (BAC92733.1) Hd1-like protein, respectively. The observed SNP differences between our lines translate into one amino acid (leucine to phenylalanine) difference at position 169 of the wheat protein of 370 amino acids.

The next step after identifying the sequences of *ScHd1* and SNP was the design of new primers to identify the C-T mutation via PCR. The same forward primer was used as previously for amplification, together with one of two new reverse primers: Hd-R1-312R19 (5-´ACATATTGTATCCGACAAA-3´) or Hd-R2-312R19 (5´-ACATATTGTATCCGACAAG-3´) together with hot-start VivaTaq polymerase (Novazym).

The SNP polymorphism in the analysed lines was confirmed using allospecific primers for two variants of the *ScHd1* sequence and the analysis was also performed for two mapping populations: S120×S76 (RIL-M) and C599×620-2 (RIL-R). A single gene segregation (*χ* ^*2*^ = 1.29) was obtained for the first population, whereas the allelic polymorphism of the *ScHd1* fragment was not found in the second population. The inbred line C599-5 analysed in this experiment was a sister sub-line with respect to that used to produce hybrid C599×620-2. These sub-lines must have differed in terms of mutations in *ScHd1*. Segregation data for the RIL-M population was added to data from previous analyses, mainly using DarT markers (Myśków 2012; Myśków et al. 2012); mapping of *ScHd1* was performed via JoinMap 3.0 software (Van Ooijen and Voorrips 2001) and the marker was associated with loci on chromosome 6R. Mapping was executed for a group consisting of 44 markers most significantly associated with *ScHd1* (LOD = 15). The map position of the gene and its nearest loci is presented in Fig. 1.

**Fig. 1:**
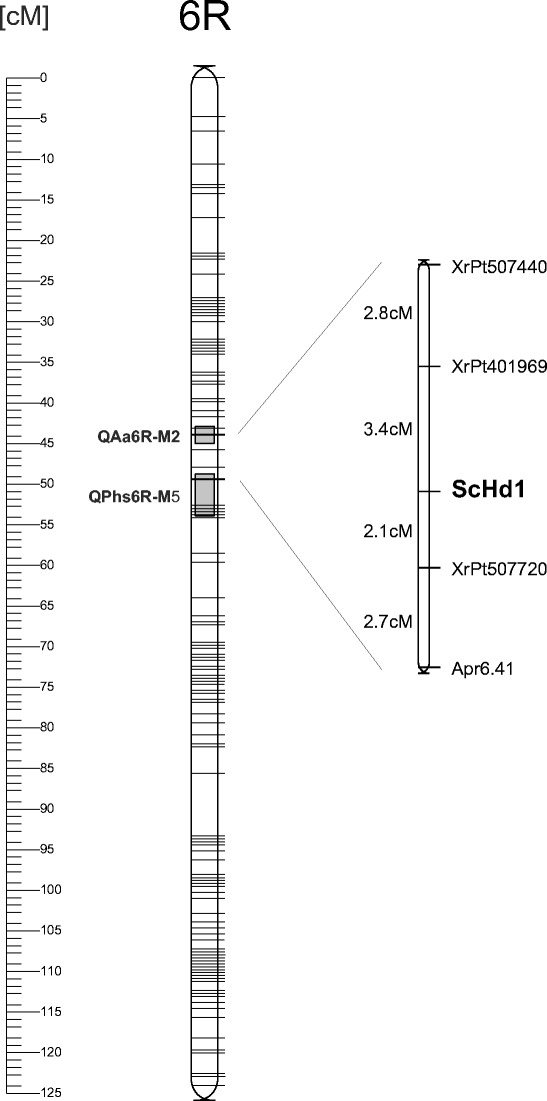
Localisation of *ScHd1* on the genetic map of rye on chromosome 6R in the RIL-M population. Grey rectangles mark QTLs mapped to the close vicinity of the gene (QTL for α-amylase activity and pre-harvest sprouting)

The sequence analysis and the map position suggest that *ScHd1* is homologous to the *CO*/*Hd1* gene. *TaHd1* was located on the long arm of chromosomes belonging to group 6 in hexaploid wheat (Nemoto et al. 2003). A rye gene was also mapped to the pericentromeric region of chromosome 6, with a genetic distance of 5cM, above the RAPD – APR6.41 marker localised to the short arm on the integrated 6R map (Stojałowski et al. 2009). In the light of new data concerning rearrangements of the rye genome in comparison to wheat (Li et al. 2013), the short arm 6R corresponds not only to the short arm of wheat chromosome 6, which was previously postulated (Devos et al. 1993), but also includes fragment of the long arm in the centromeric region. Therefore, localisation of *ScHd1* to the short arm is consistent with present knowledge about genome synteny.

A map used for the map position of *Hd1* in rye was previously used for the localisation of the eps loci and of QTLs for different morphological traits such as PHS, AA, Ph, Sl, Sps, Kps, Kw and Tkw (Myśków et al. 2012, 2014). Additionally, the spike density coefficient (Ct) was calculated from the formula: 10 cm × Sps/Sl and collected data were included for further analysis. The results enabled a relationship between *ScHd1* segregation and the distribution of the above-mentioned traits in the mapping population to be detected. The Kruskal*–*Wallis test (STATISTICA 9.0, http://www.statsoft.com) was used to determine inter-relationships and the results are depicted in Table 1. It was also assessed whether the gene co-locates with known QTLs.

**Table 1:** Relationship between the segregation of *ScHd1* alleles and heading earliness and different morphological traits in the rye mapping population RIL-M (143 inbred lines), identified using the Kruskal-Wallis test (only statistically significant). He – heading earliness (on a nine-point scale), Sl – spike length (cm), Kw – kernel weight per spike (g), Tkw – thousand kernel weight (g), Ct – density of spikelets (number of spikelets per 10 cm of spike). Measurements of earliness were made for four years (2007–2010); other traits were measured over 3 years (2008–2010)

Trait	N number of lines	H Kruskal-Walis test statistic		Mean value of trait		
			P probability	Allele C (S120)	Allele T (S76)	Difference
He/07	104	6.64	0.01	4.75	5.91	1.16
He/08	131	6.56	0.01	4.33	5.06	0.73
Sl/08	132	6.73	0.01	8.06	8.56	0.50
Sl/09	132	4.06	0.04	6.70	6.99	0.29
Sl/08–10	131	5.25	0.02	7.45	7.83	0.38
Kw/08–10	131	5.48	0.02	0.56	0.66	0.10
Tkw/08	132	4.04	0.04	26.02	28.08	2.06
Tkw/10	124	5.01	0.03	19.37	20.47	1.10
Tkw/08–10	131	6.41	0.01	22.99	24.75	1.76
Ct/08	132	11.03	0.00	26.69	28.33	1.64
Ct/09	132	3.75	0.05	27.36	28.31	0.95

One or two QTLs for earliness per se, depending on the mapping population, were observed on chromosome 6R (Stojałowski and Łapiński 2002; Myśków 2012; Myśków et al. 2012). *ScHd1* was not mapped to any of the two QTLs present in the RIL-M population map (Myśków 2012; Myśków et al. 2012). However, the relationship of this marker with the phenotypic segregation of eps was shown to be statistically significant in two years out of a four-year research study, using the Kruskal–Wallis test. The non-parametric Kruskal–Wallis test frequently leads to different results than the CIM analysis (Myśków et al. 2014) and is less accurate with respect to QTL mapping; however, it is a simple method of marker identification that can be used for the selection of an evaluated trait. It also enables the evaluation of a phenotypic effect that can be obtained using a certain selection marker. The Kruskal–Wallis test showed that *ScHd1* can affect heading by about one point on a rating scale (according to Masojć and Milczarski 1999), which corresponds to approximately 1 day. Heading earliness was associated with the presence of an allele from line S76. Although the line S76 is later compared to S120, transgression into the RIL-M population and QTL analysis indicates the presence of many eps genes derived from line S76 (Myśków et al. 2012).

The presence of pleiotropic effects is an interesting phenomenon with respect to genes that regulate flowering time. Numerous studies concerning *Ppd* and *Vrn* genes have confirmed their simultaneous effect on other vital plant traits. Pleiotropic effects of *Ppd* genes on plant height, spike morphology, and the number and weight of kernels has been confirmed in wheat (Worland et al. 1998) and barley (Laurie et al. 1994; Karsai et al. 1999; Wang et al. 2010).

Several studies have also suggested a relationship between the activity of intrinsic earliness genes and other important crop traits. For example, it was established that the best-characterised gene of earliness per se (locus *Eps-A* ^*m*^ *1*) also exhibits pleiotropic effects and affected the number of spikelets per spike of the diploid wheat via regulation of the length of the spike development stages (Lewis et al. 2008). In diploid wheat, it was also observed that the QTL for the heading date from 3A overlapped with a QTL for the length of spike and number of spikelets. The co-localisation of some QTLs of earliness per se with QTLs for pre-harvest sprouting was observed in rye (Myśków 2012; Myśków et al. 2012), which might indirectly provide evidence concerning the influence of flowering time genes on the germination process in subsequent progeny.

The results presented here show that similar to other wheat and barley eps genes, *ScHd1* might be related to morphological traits, including spike morphology, such as the length of the spike, spike density and thousand kernel weight (Table 1). Plants with the allele from line S76 were also characterised by earlier heading, an elongated spike, an increase in the number of spikelets *per* spike, increased weight of kernels *per* spike and thousand kernel weight. This might indicate that the confidence interval of an average QTL usually contains several dozens of polymorphic genes for different features.

Throughout the three years of study, the Kruskal–Wallis test showed no correlation of *ScHd1* segregation with α-amylase activity or susceptibility to pre-harvest sprouting; however, this gene is linked with the XrPt507440 and XrPt507720 markers that localise within QTLs responsible for these traits.
